# Infant Feeding: Proceedings I. H. A.

**Published:** 1892-02

**Authors:** 


### INFANT FEEDING.

Proceedings I. H. A., Wednesday morning, June 24th.

A paper entitled "Indigestion in Infants," by Dr. Nathan Cash, Uhrichsville, Ohio, was read. This paper can be found in full in THE HOMŒOPATHIC PHYSICIAN for October, 1891, page 394. The following discussion ensued:

Dr. Custis—This subject is one in which I am very much interested. As a class, we have the means of being the most independent of physicians, because we have a law of therapeutics leaving us free to use any other law of chemistry, physics, or mechanics that may aid us. The doctor tells us that milk is the proper food for babies, also that the milk should be brought directly from the cow to the baby, and that the cow should be healthy and well kept, and so on. This is all very well, but it is impossible to the vast majority of patients. Cows cannot be kept in cities; if they are, they will probably be unhealthy. The milk cannot be brought to the city perfectly fresh. The patient can know nothing of the history of the particular cow or set of cows that supplies her. Now what are we going to do about it? We must go to the laws of physiological chemistry and prepare the milk so, as much as possible, to restore it to its perfect condition. The artificial foods are made by competent scientific men, according to the laws of chemistry and physiology. I find very few babies, in my experience, who can take cow's milk plain. They cannot assimilate it. Ninety-nine out of a hundred cases of infantile indigestion come from improper feeding. If the child sleeps well, then you are feeding it pretty nearly right; but if it does not sleep well, no matter what your food, you are feeding it improperly. Then you must help it by artificial means. If the child shows symptoms of sickness, fever, forcible vomiting, etc., it needs medicine, but if it shows no evidence of special sickness, and does not sleep well, and the stools are not perfectly normal, something is the matter with the food. Mellin's food is one of the most valuable foods in the market. Imperial Granum is good for little children. The study given to the subject by the men who make the foods is something remarkable; they can give us points on digestion every time. Foods are not medicine, and do not interfere at all with our therapeutics.

Dr. Kent—The essayist strikes at the vital point of everything that the physician has to deal with, when he speaks of infants and indigestion. So much is to be said on this subject that there is hardly a beginning and I am sure no ending. The first critic of the essay wants to be called a scientific physician; I do not. I do not want to be known as scientific. A man who is pointed out as scientific has pretty nearly reached the height of folly. We might divide infants into two classes—those who have the power to live, and those who have the power to die. The next division is the sick child and the natural child. If born with a sick miasm, he needs medicine. The next class is the natural child who has been made sick by food, and this is one of the most prominent things which we have to meet. This is the starting-point of the paper. As to the food, if the infant is in a natural state, we know that milk is its natural food. But when it becomes spoiled by improper feeding, or over-feeding, the child becomes unnatural.

If the child is natural and the milk is natural we have no trouble. But even when both are natural too frequent feeding will cause indigestion, known first by lienteric stools.

This goes on until the question has become a common one, "Why is it that the second summer is so hard for babies?" and we all know the answer. I often put the question to the housewife, "What would happen if, when the sponge for bread was half raised, you were to put flour in?" It would sour at once, and in the same way the stomach sours from the frequent feeding. The weakly ones will stand this until the first hot weather comes; the tough ones until the second summer, and then they come down with cholera infantum.

The natural infant, fed on milk, gets on the best; but when fed on milk unwisely until they can no longer digest it, we are compelled to change off to some other food for awhile. As soon as the stomach has been corrected by natural and regular feeding we can go back to milk again. A young baby should be fed at intervals of two hours, and not at all during the night. In artificial foods you have to use experiments, and after several trials some one of the many chemical foods will serve a useful purpose.

I am frequently asked "Why not use peptonized milk?" It is improper to use the digested preparations because, as soon as you furnish the stomach with Pepsin it ceases to make its own Pepsin; you encourage it in laziness. If the child is vital the remedies are sufficient. It is a pretty poor infant that will not drink water. Infants passing lienteric stools can generally drink water, if they cannot, they need medicine. In such cases I am in the habit of diluting the milk pretty well with water, and when the stool is improved, going back to stronger food.

Dr. MacLaren—I think we must individualize in the matter of foods as well as in the matter of medicines. In Guernsey's work on obstetrics, there is a preparation recommended that will save lots of babies. It is one-third cream, one-third water, one-third sugar of milk. When a baby is sick and the mother has no milk, I hunt for a wet-nurse. I had an interesting case ten months ago. A healthy child was born of a weakly mother. She had lost four children before; she had decided symptoms of phthisis. I used this food of Dr. Guernsey's, the child relished it, and it agreed with it. The baby slept well, seemed well nourished, and ate very little at night; during the day it got a meal every two hours. In this way four weeks passed without a remedy being required, but it did not seem to grow a bit. At the end of that time we got a wet-nurse, and it soon weighed twenty-five pounds.

Dr. Wesselhœft—A very important thing is the way the milk goes down into the child's stomach. The bottles are so constructed that the milk goes down too fast without being mixed with the saliva. Every child who sucks at the breast has to work for what it gets, and this suction brings the salivary glands into operation, and I fear that that is one of the great troubles in artificial feeding. The milk is cascaded into the stomach and immediately cascaded back again. I have always in my practice acted on the principle that a good cow is better than a wet-nurse, for my own use I would much rather have a cow than a wet-nurse. As regards the milk being too strong or too weak for the baby, it seems nonsense to me. Any little animal will bear cow's milk perfectly well, and if you could get enough milk from a mouse you could raise an elephant on it. It is astonishing how little milk a healthy baby can get along with during the first week of its life, and the amount should be only very gradually increased. Most of the sick babies are made so by some prepared stuff being cascaded into their stomach in enormous quantities. Quantity is a great element in these disorders, and I have known too much food to make babies sick, even when the food was perfectly fresh milk, and the babies did their own milking.

Dr. Biegler—This is a matter in which I feel some interest, because I have necessarily had to give a great deal of attention to it. I agree with Dr. Wesselhœft that rapid feeding is bad. The bottle arrangements are made so that the milk comes down too rapidly, and in great quantities. I generally tell the mother to put a piece of pure clean sponge into the nipple so that the child must work with its gums and lips to draw the milk, and thus obviate the too rapid flow. The strength of the milk should be regulated I think for new-born infants. The best substitute for mother's milk is cow's milk. The artificial foods in my experience are all valueless. Of course the cow or cows should be healthy, and a very important point is the food of the cow. Let a child take milk from a cow that yesterday ate green apples, and that child will be sick as certainly as the sun will rise. We have to depend upon the milkman in cities, and the cows are often fed in a horrible manner. The refuse from glucose factories, sour, stinking, and offensive, is the food of many cows, and of course such milk is injurious.

In regard to the strength of milk we must discriminate. In nine cases out of ten, milk from a Jersey herd will not be well borne by children. There is no inflexible rule suitable for all infants. Find out what each individual infant can digest I have seen infants progress from a deplorable state to a most chubby and beautiful condition without ever tasting milk of full strength.

Dr. J. V. Allen—I do not agree with the position that the milk should be taken slowly in order to have it mix with the baby's saliva, for the reason that the saliva enters into no digestive process whatever with the infant. The child must have teeth before it has any saliva. Whether the child eats slow or fast the saliva has no action on the milk.

Dr. Biegler—In the natural way, does the milk pour down the child's throat from the mother's breast, or is it slowly drawn out?

Dr. J. V. Allen—I believe a little exercise is very good, but as far as the digestion of milk goes, it cuts no figure.

Dr. Wesselhœft—Is there no saliva in the baby? What is it makes his mouth moist?

Dr. J. V. Allen—Only mucus.

Dr. Wesselhœft—Let us have mucus, then, mixed with the milk.

Dr. Hastings—I have used with success what is called evaporated cream—a preparation known as the Highland Brand of condensed milk. It is milk from well-fed Jersey cows, carefully evaporated to the consistency of first-class cream, and entirely free from extra sugar or other artificial additions. It is then, while still warm, hermetically sealed and makes, I think, a very fine substitute for mother's milk. I have a little patient who makes a very fine showing upon it, and, although born at the twenty-seventh week, has never had a day's sickness. I know of two other children, both doing well on this food. Of course it is used diluted. It is essentially milk with a portion of the water evaporated; hence it can be restored to the condition of fresh milk by diluting it.

Dr. Dever—Three cases are not sufficient to prove anything by.

Dr. Hastings—I did not speak of anything being proved. I was simply giving my mite of experience.

Dr. Biegler—Jersey cows are thought to produce the finest milk, but I have a word to say on that subject. In my capacity as member of the Board of Health I have had a great deal to do with the milk business, and in that work I ascertained the fact that Jersey cows are apt to be tuberculous, and are often on that account quietly killed by their owners, whenever it becomes their interest to do so. In this way numbers of cows are quietly killed and nothing said about it. What we know about the tuberculous diathesis in man would lead us to suppose that this is true, for these cows possess the same or similar characteristics, namely, a clear and beautiful complexion, a thin skin, and a large transparent eye. On this account I should prefer milk from some other breed, such as the Holstein.

Dr. Stow—The question of the rearing of infants has caused me as much perplexity as any I have had to do with. The use of nursing bottles, to be successful, requires the constant, intelligent care of the nurse or mother, to keep them perfectly clean and free from impurities, especially during the summer months. In warm weather milk ferments very rapidly, and I think nursing bottles should be generally discarded until we get some better material to make the fittings of than rubber. I think that in every city in the land organizations should be formed to provide pure fresh milk for infants. It should come not from Jersey cows but from Guernseys or Holsteins, placed into scoured cans and kept at a low temperature in refrigerator-wagons for dispensing to customers. This has been tried in some places, and wherever it has been tried it has proved a success.

Dr. Rushmore—Microscopic examinations have shown the milk of the Ayrshire to be better than any other breed.

Dr. Sawyer—I have succeeded pretty well with children as a rule, but once or twice when a little patient died suddenly I have traced it to the abominable water which the cow had to drink. I believe in artificial foods emphatically. I am in favor of them in a great many cases. Horlick's food is sometimes very valuable. I have raised babies on this food which I know positively would not have lived on mother's milk. I have tried all kinds of cows, single and married, virtuous and rakes, one cow and cumulative, and generally prefer the foods. I have had to banish the miserable nursing bottles entirely. I prefer giving the milk or food in small quantities from a cup.

Dr. W. L. Morgan—I have had considerable experience with several kinds of artificial foods, and I like them very well. I I never use any of those that have Pepsin in them or that are pre-digested.

Dr. Custis.—The stomach in its function of digesting food does perform certain chemical processes which it is just as much our business to study scientifically as it is to study the action of remedies, for if we knew all about it we could prevent sickness, and thus have no occasion to use our remedies. I am glad Dr. Hastings spoke of that Highland Brand of milk for I am going to try it.

Dr. N. Cash—We may become too chemically scientific in discussing this question. My stand would be that as soon as milk cools and loses its animal heat, chemical changes begin.

